# Measuring volume fractions of a three-phase flow without separation utilizing an approach based on artificial intelligence and capacitive sensors

**DOI:** 10.1371/journal.pone.0301437

**Published:** 2024-05-16

**Authors:** Abdulilah Mohammad Mayet, Farhad Fouladinia, Seyed Mehdi Alizadeh, Hala H. Alhashim, John William Grimaldo Guerrero, Hassen Loukil, Muneer Parayangat, Ehsan Nazemi, Neeraj Kumar Shukla

**Affiliations:** 1 Electrical Engineering Department, King Khalid University, Abha, Saudi Arabia; 2 Faculty of Engineering, Rzeszow University of Technology, Rzeszow, Poland; 3 Petroleum Engineering Department, Australian University, West Mishref, Kuwait; 4 Department of Physics, College of Science, Imam Abdulrahman Bin Faisal University, Dammam, Saudi Arabia; 5 Department of Energy, Universidad de la Costa, Barranquilla, Colombia; 6 Faculty of Engineering, University of Southampton, Southampton, United Kingdom; DIT University, INDIA

## Abstract

Many different kind of fluids in a wide variety of industries exist, such as two-phase and three-phase. Various combinations of them can be expected and gas-oil-water is one of the most common flows. Measuring the volume fraction of phases without separation is vital in many aspects, one of which is financial issues. Many methods are utilized to ascertain the volumetric proportion of each phase. Sensors based on measuring capacity are so popular because this kind of sensor operates seamlessly and autonomously without necessitating any form of segregation or disruption for measuring in the process. Besides, at the present moment, Artificial intelligence (AI) can be nominated as the most useful tool in several fields, and metering is no exception. Also, three main type of regimes can be found which are annular, stratified, and homogeneous. In this paper, volume fractions in a gas-oil-water three-phase homogeneous regime are measured. To accomplish this objective, an Artificial Neural Network (ANN) and a capacitance-based sensor are utilized. To train the presented network, an optimized sensor was implemented in the COMSOL Multiphysics software and after doing a lot of simulations, 231 different data are produced. Among all obtained results, 70 percent of them (161 data) are awarded to the train data, and the rest of them (70 data) are considered for the test data. This investigation proposes a new intelligent metering system based on the Multilayer Perceptron network (MLP) that can estimate a three-phase water-oil-gas fluid’s water volume fraction precisely with a very low error. The obtained Mean Absolute Error (MAE) is equal to 1.66. This dedicates the presented predicting method’s considerable accuracy. Moreover, this study was confined to homogeneous regime and cannot measure void fractions of other fluid types and this can be considered for future works. Besides, temperature and pressure changes which highly temper relative permittivity and density of the liquid inside the pipe can be considered for another future idea.

## 1. Introduction

Multiphase flows are common in numerous industries, including petrochemicals, gas, oil, and so on. A fluid of a mixture of solid, liquid, or gas phases, known as multiphase flow, is frequently encountered in various industrial production processes and finds application in power generation, the industry of chemicals, oil extraction, nuclear energy, and metallurgical engineering.

Diverse mechanical and electrical methods are extensively utilized to observe various flow patterns. Traditionally, the approach, taken to address the challenge of measuring multiphase flows, involves the initial separation of a fluid’s components followed by the individual and the intricately challenging task involves quantifying the flow rate of each component by employing conventional instruments designed for single-phase fluid measurements [1].

Some of mechanical techniques utilized for such a task are sampling tubes, turbo meters, vortex meters, and vibrating densitometers can be mentioned. However, a limitation of these methods lies in their intrusive nature, as they necessitate the measurement of some characteristics using sophisticated devices [2, 3].

A three-phase fluid which involves the mixing of gas, oil, and water, usually exists during petroleum transportation. As a result, the essentiality of continuously monitoring volume fractions in this kind of three-phase fluid becomes evident when it comes to logging and metering pipelines that transport petroleum.

Despite having been the subject of intensive worldwide research and development for over three decades, the precise quantification of the flow of oil-gas-water blends within a pipeline continues to be regarded as one of the primary hurdles encountered in the petroleum sector [4]. To solve this critical issue as much as possible, a myriad of methodologies have been harnessed to ascertain the volumetric proportion of three-component fluids, with the prevalent approaches encompassing the utilization of gamma-ray attenuation and capacitance-based sensors.

On the one hand, gamma radiation techniques have been the focus of numerous studies to quantify the volumetric ratios of gas, oil, and water. Among all previous studies, a number of them can be found that their main focus was on a particular fluid. The investigated fluid in this study is a three-phase homogeneous one that contains water, oil and gas. In [5], Salgado and his colleagues pioneered the introduction of a state-of-the-art system using dual-energy gamma emitter radioisotopes, delving into the intricacies of gas-oil-water multiphase flow, an intricate pursuit revolves around the discernment and identification of distinct flow regimes.

On the other hand, in the realm of popular electrical techniques, for over two decades, Electrical Capacitance Tomography (ECT) has been pursued by researchers as a promising imaging method [6, 7]. Over the past decades, researchers have devised an extensive array of monitoring methods to tackle the intricate randomness and complex nature of fluids [8, 9]. Although ECT generally produces satisfactory images for two-phase flows, as the number of phases increases, the reconstruction of images using ECT measurements becomes progressively more challenging [10]. In a recent development within tomographic methods, a technique known as the One Excitation for Simultaneous High-speed Operation Tomography (ONE-SHOT) was introduced in [11]. The primary objective was to capture images of multiphase flows with rapid frame rates. The approach involved simultaneously applying electrical excitations and driving overlapping measuring signals, distinguished through FFT analysis. The system successfully produced images at a rate of 3906 frames per second, showcasing a significant advancement in comparison to previous tomographic methods.

The calculation of the dielectric properties of the liquid was frequently accomplished by analyzing measured inductance, capacitance, or resistance which resulted in measuring the volume fractions [12, 13]. For instance, in [14] the flow meter based on correlation was utilized to determine water content. To achieve this goal, conductance measurement was employed for the three-phase mixture. Additionally, reference [15] introduced a capacitive sensor with multiple electrodes for monitoring of two-phase fluids, which necessitated microcontroller analysis. Other studies have been conducted in this subject. Authors in [16], to enhance the variety of imitated phase distributions, simulations using a particular electrode configuration performed, such as the finite element method (FEM). Similar to simulations based on experiments, curves fitted to describe the relationships between capacitance and some of relevant parameters. Furthermore, the Texas Instruments FDC2x1x-series is an integrated circuit based on an LC-tank. The IC’s digital value signifies the resonant frequency of the LC-tank circuit, utilized for calculating the equivalent measured capacitance. Stray capacitances are considered as an offset in the measured values, and with appropriate calibration measurements, they can be disregarded. This IC has been incorporated into a prototype device for assessing gas-liquid flow in potentially dangerous environments and estimating the void fraction of two-phase flows in tube bundles [17, 18]. In their study [19], investigators employed a PCap04 to gauge water level and quality, relying on an interdigitated sensor design. The PCap04 provided a method for measuring capacitance and carrying out on-chip data processing by combining a CDC with a digital signal processor (DSP). The sensing approach gauged the time it took for the capacitance to charge and discharge. In the investigation by Yang and colleagues [20], they applied a capacitance sensor with parallel plates to determine the mass flow rate of a two-phase flow involving gas and solid particles. The sensor segment had dimensions of 2 meters in length and 0.5 meters in width. Moreover, in another study, wire-mesh sensors were employed to measure gas-liquid-liquid flows, showcasing the sensor’s ability to study three-phase flow with some success, albeit limited as only one electrical parameter was measured. This limitation has been addressed by advancements in the dual-modality wire-mesh sensor [21] enhancing the accuracy of measurement by measuring both resistance and capacitance values.

In the realm of multiphase flows, the joint simulation involving Finite Element Method (FEM) holds promise. In the foreseeable future, this approach could generate data to machine learning algorithms, rendering calibrations independent of specific fluid properties. This type of methodology aligns with the current trend, reflecting the progress of artificial intelligence in diverse processing routines across various domains in recent years [22]. Recently, a lot of investigations have done utilizing capacitance-based sensors and an Artificial Neural Network (ANN) to quantify volume fractions in a dual-phase fluid. In a compelling exploration undertaken by authors in [23], the effectiveness of an advanced radial basis function (RBF) neural network and the cutting-edge method based on gamma transmission were thoroughly examined for prognosticating volume fractions about the water-oil-gas annular fluid. Three distinct RBF models were explored, the first, second, and third models being utilized for estimating water and oil volumetric proportion, gas and water volumetric proportion and gas and oil volumetric proportion, respectively. The results indicated that the first RBF model exhibited superior precision in estimating volumetric proportions in the three-phase annular fluid. Moreover, a meticulously constructed MLP model was devised with the capability to precisely prognosticate volume fractions within a two-phase annular fluid [24]. Also, an MLP ANN was modeled [25] to measure volumetric proportion in a homogeneous fluid regardless of the specific liquid phase composition. It can be seen that a substantial surge in popularity is witnessed in the expansion of ANN as an instrument in the expansive realms of electrical engineering and control engineering. Consequently, in the contemporary scientific inquiry, recent studies have embarked on a rigorous exploration of the optimization pathways for sensors. This endeavor involves leveraging the immense potential of ANN tools and harnessing their inherent capabilities to uncover novel strategies and methodologies that can augment sensor outcomes, significantly [26, 27]. Besides, in [28], an 8-electrode sensor based on capacity was utilized to predict the void fraction of a two-phase homogeneous fluid independent of variations in the pressure. To achieve this goal, an MLP ANN was employed. In addition, in [29], a method that combined dual-energy gamma attenuation with an MLP ANN was suggested for the concurrent identification and quantification of four distinct petroleum by-products. Authors in [30], the study investigated the possibility of utilizing an X-ray tube in place of radioisotope sources to assess the volume fractions of gas, oil, and water within two common flow patterns, annular and stratified. Furthermore, the acquired data was analyzed using the potent regression tool known as the Group Method of Data Handling (GMDH). Last but not least, Gholipour Peyvandi and associates in [31] utilized a combination of a gamma backscatter technique and an MLP neural network to make estimations regarding the volume fractions of gas, oil, and water.

In this paper, a novel metering system has been presented that can measure phases separately. The water volumetric proportion in a three-phase homogeneous fluid containing gas, oil, and water is measured in this article. To accomplish this objective, employing state-of-the-art methodologies and advanced techniques was necessary. So, a capacitance-based sensor and an ANN are utilized. To train the presented network, an optimized sensor is applied in one of the most useful software, the COMSOL Multiphysics software, and a lot of simulations are done. This investigation proposes a new intelligent metering system based on the Multilayer Perceptron network (MLP) that can estimate a three-phase water-oil-gas fluid’s water volume fraction precisely with a very low error. The obtained Mean Absolute Error (MAE) is equal to 1.66. This dedicates the presented predicting method’s considerable accuracy.

In the following, the utilization of the aforementioned software and implementation of the optimized capacitances-based sensor in this software are presented in section 2. In the subsequent stage, in section 3, the proposed model is introduced in detail. After that, the obtained results are given in section 4. Finally, in section 5, the paper is concluded.

## 2. Methodology

Capacitance-based sensors are non-invasive and do not require direct contact with the substance. Hence, in this paper, a kind of the mentioned sensor is utilized. First, two of the most important physical characteristics of materials are introduced. Density is a measure of mass per unit volume which describes the compactness of matter in a substance, whereas relative permittivity is a measure of how well a material can store electrical energy in an electric field compared to free space, and as mentioned before, both properties play vital roles in various scientific and engineering applications. As mentioned, many methods are utilized to measure volume fractions but two of them, gamma-ray and capacitance-based sensors, are the most popular ones. The fundamental element in the gamma-ray method is ray attenuation which relies on measuring the attenuation of gamma rays as they pass through a flowing material, such as a liquid or gas. When gamma rays interact with matter, they can undergo different processes depending on the energy of the ray and the type of material they pass through. The three primary interactions of gamma rays with matter are Compton scattering, photoelectric absorption, and pair production. So, it can be taken that this method highly depends on the density of the matter. Therefore, materials with higher density tend to have more atoms per volume’s unit which increases the likelihood of gamma rays interacting with the material as they pass through it. This higher interaction probability leads to increased gamma-ray attenuation. As a result, fewer gamma rays reach the gamma-ray detector on the other side of the material which results in a lower gamma count. On the other hand, materials with lower density have fewer atoms per volume’s unit which leads to fewer interactions with gamma rays. Consequently, these materials cause less attenuation of gamma rays, and more gamma rays reach the detector resulting in a higher gamma count. The absorption law as Eq (1) is a mathematical correlation between the initial intensity of γ-rays (I_0_) emitted by the γ-ray source and the intensity that remains (I) after passing through a thickness L of an oil/water/gas (L) and density (ρ) which is given as below [32]:

$$I(E)=I_{0}(E)exp(‐\mu (E).L)$$ (1)

$$\mu (E)=\alpha .\mu {\left(E\right)}_{o}+\beta .\mu {\left(E\right)}_{w}+\gamma .\mu {\left(E\right)}_{g}$$ (2)

where in Eq (2), μ(E)_o_, μ(E)_w_, and μ(E)_g_ are the linear attenuation coefficients of the oil, water, and gas phases, respectively. Also, α, β, γ are the volumetric fractions related to the aforementioned matters in a similar order. On the other hand, μ(E) = η(ZE)ρ, the absorption coefficient η is a function of the γ-ray energy (E) and the atom number (Z).

In capacitance-based sensors, as it is clear from their name, the main characteristic of this kind of sensor is the measured capacity. The capacitance (C) of a capacitor is directly proportional to the relative permittivity (ε_r_) of the dielectric material between the capacitor plates as well as to the area (A) of the plates and inversely proportional to the distance (D) between the plates. The relationship can be expressed using the Eq (3) introduced in [33]:

$$C=\varepsilon_{r}*\varepsilon_{0}*A/D$$ (3)

Where ε_0_ = Permittivity of free space (approximately 8.854 x 10^-12 F/m)

**Fig 1:**
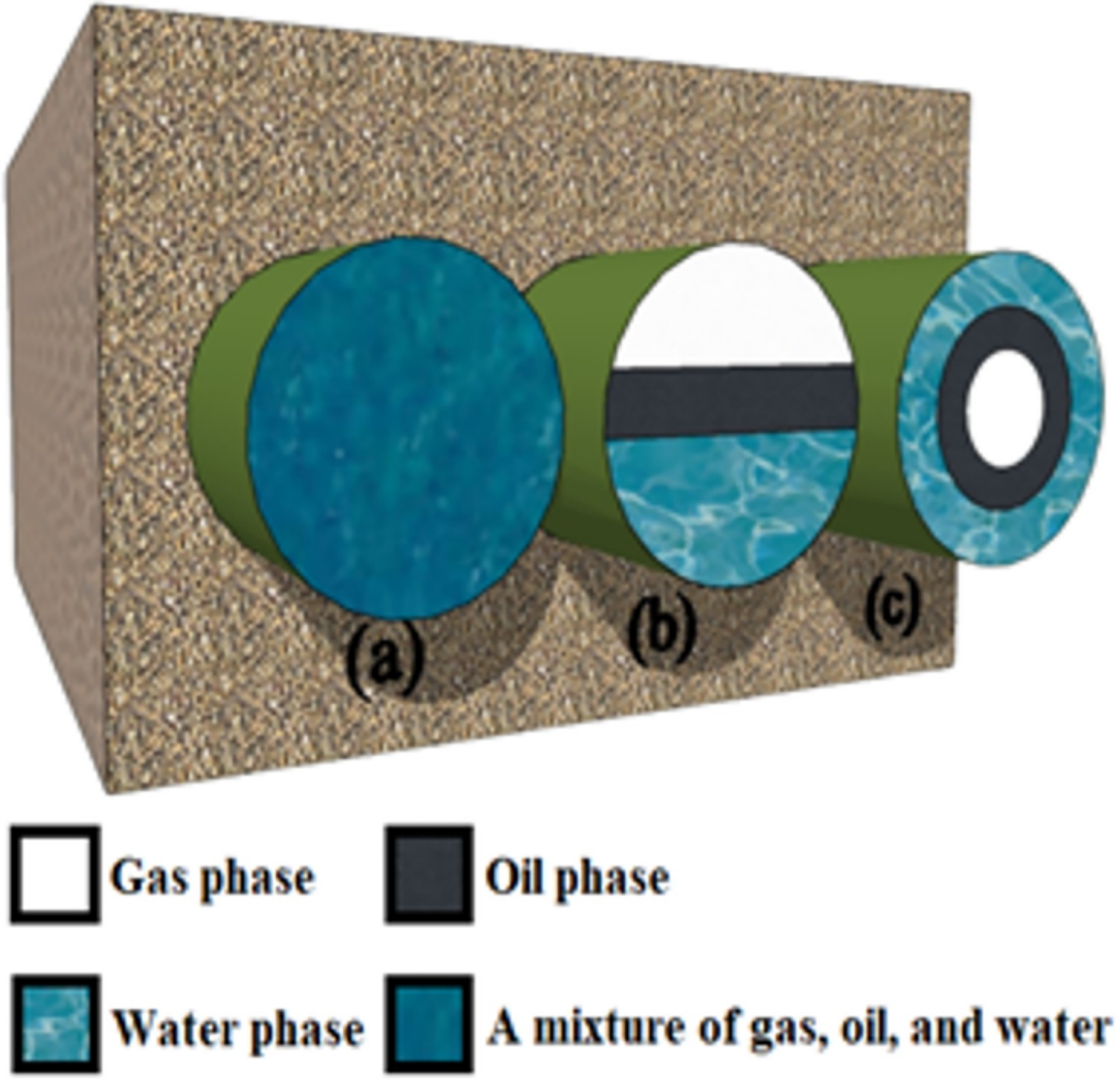
Gas-water-oil three-phase fluid different regimes. (a) homogeneous (b) stratified (c) annular.

In this paper, an optimized capacitance-based sensor is simulated to measure all three volume fractions and to investigate the impact of relative permittivity. This sensor which is called Twin Rectangular Fork-Like Capacitance (TRFLC), was developed by [34], and a homogeneous flow of water, oil, and gas in the COMSOL Multiphysics software is investigated by utilizing it. Recreation modeling and investigation is one of the foremost regularly utilized operations inquire about methods. Modeling and investigation make it possible to get distant a much better understanding of the framework by creating a scientific show of a system of intrigued, and watching the system’s operation in detail [35]. To opt for the best software regarding to the mentioned aspects, authors in [36] done a comprehensive study on several software and found out COMSOL at a time connecting all the material science and having a great intelligently interface appeared to be a great arrangement. A number of previous works in a wide variety of fields, particularly flow measurement, have been done that reported data from their simulations by this software [37]. Hence, this software is utilized as an authentic source to generate inputs for the proposed network. Within certain industries such as oil, petrochemical, and water, three main regimes exist, namely homogeneous, stratified, and annular fluids, as demonstrated in Fig 1A–1C correspondingly. It is to be noted that, except for the volume fractions of 0% and 100%, for other values, the measurement of each of the regimes presented in Fig 1, using different sensors leads to achieving different capacities, this figure in designed by SketchUp software. COMSOL software has been subjected to benchmarking through experimental experiments in the authors’ previous works [38]. In the mentioned reference, a pipe was made of PLA using a 3-D printer. Then, copper was chosen for the concave sensor electrodes. Finally, utilizing several straws, a homogeneous regime was made, and for measuring the capacity of the created sensor an LCR was utilized. The results obtained from the built sensor and those derived from the software displayed remarkably close correlations. This process contributed to the validation of the COMSOL Multiphysics software. Furthermore, a three-phase homogeneous regime is created when the three fluid phases—water, oil, and gas—undergo thorough mixing. To apply this particular mixture in the mentioned tool, the relative permittivity of the three portions is averaged for each volume fraction, leading to the generation of the mentioned regime.

**Fig 2:**
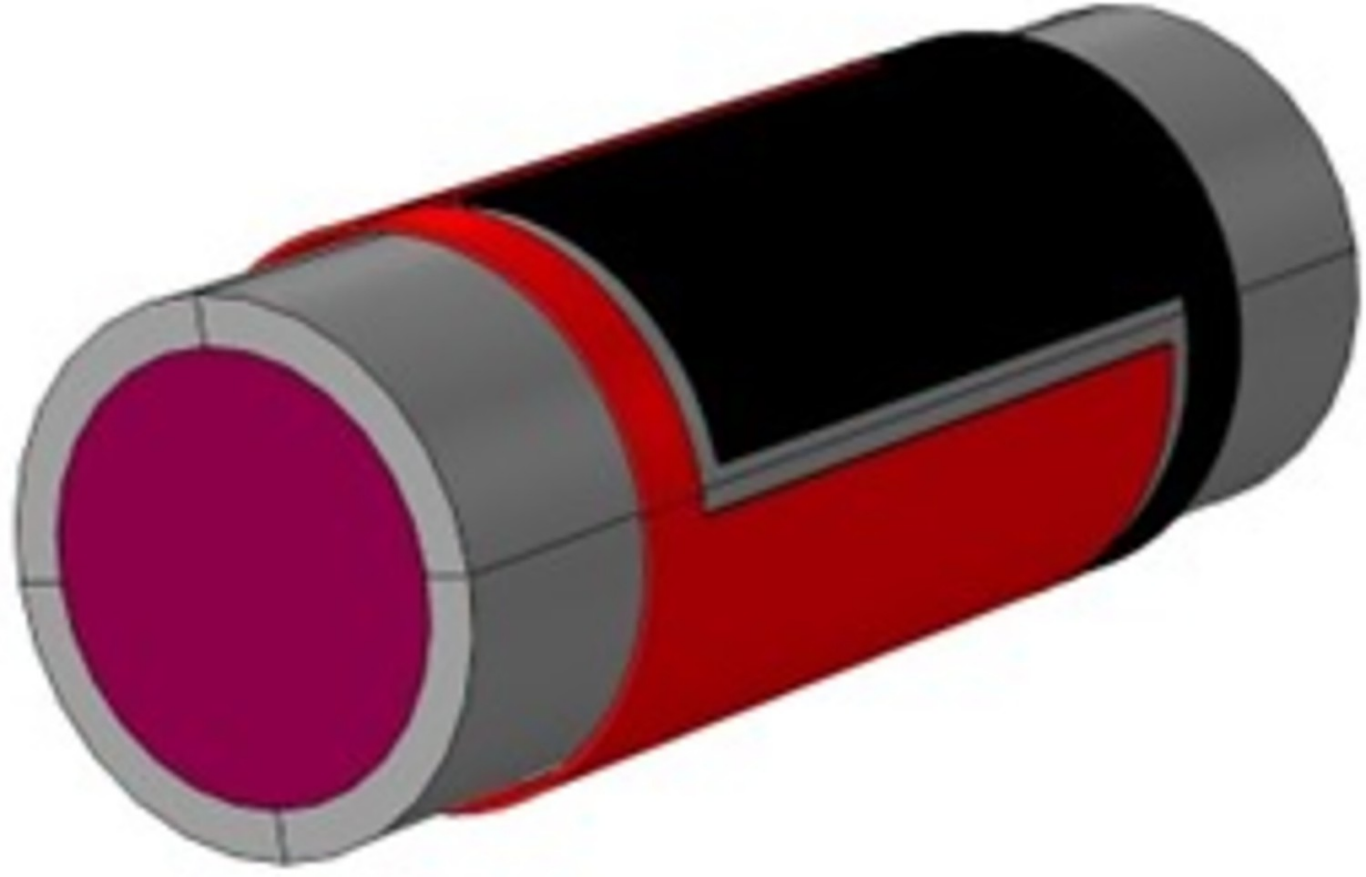
3D view of the implemented sensor.

**Fig 3:**
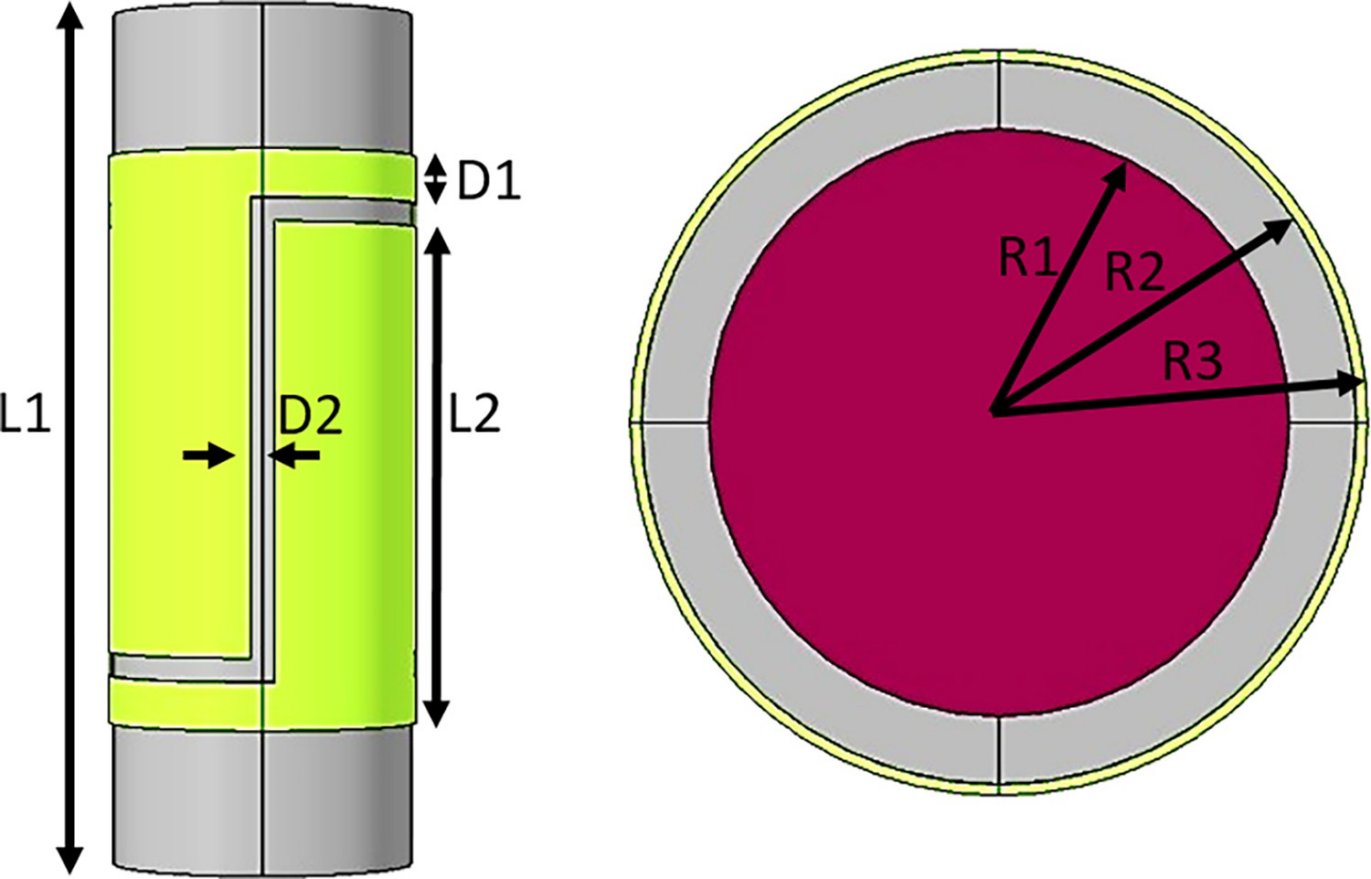
Different parts of the sensor’s sizes.

**Fig 4:**
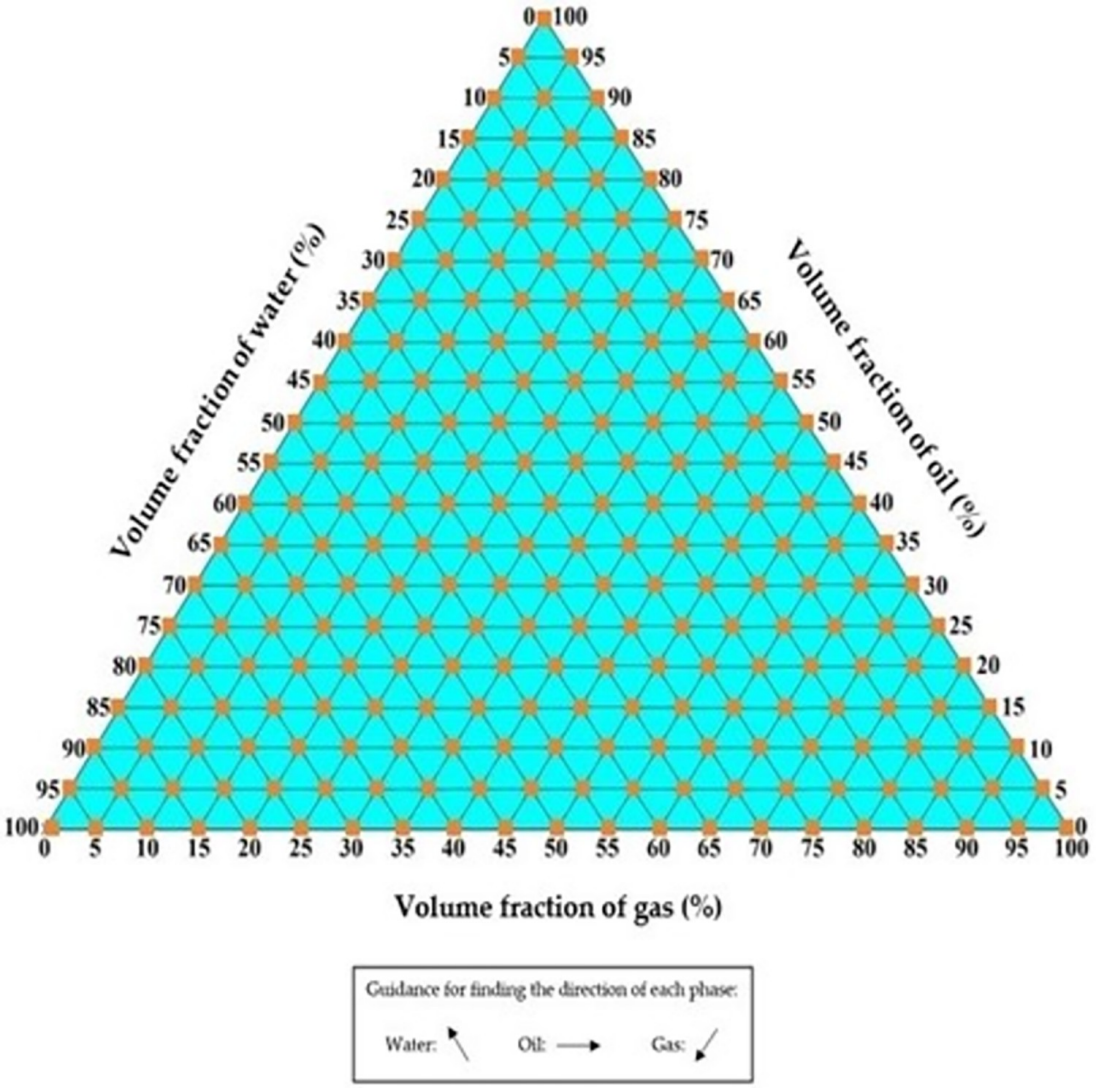
Different combinations were considered during simulations [39].

**Fig 5:**
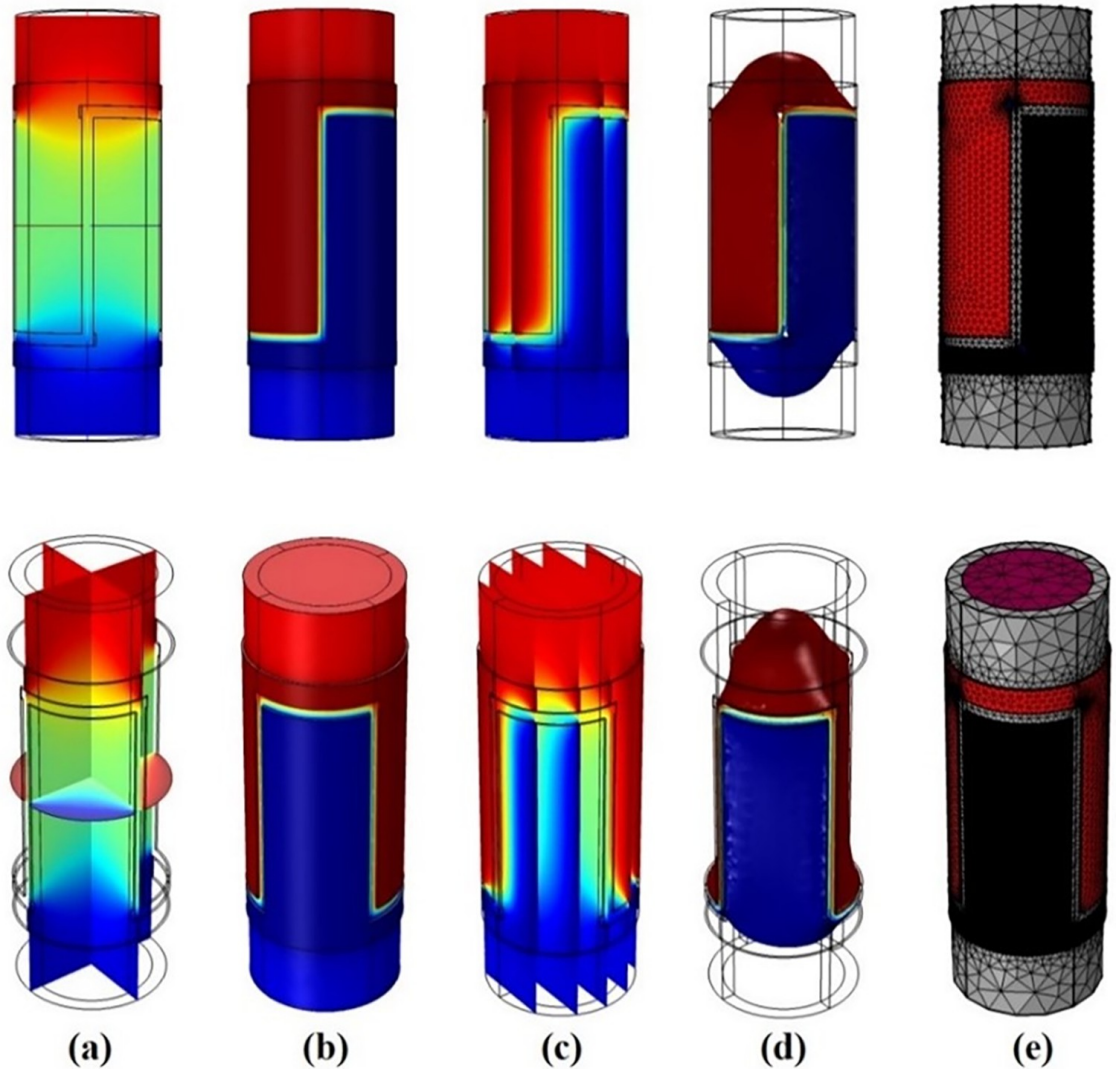
Some plots of the simulated sensor, (a) Multislice, (b) Volume, (c) Slice, (d) Isosurface, and (e) Mesh.

The TRFLC sensor is subjected to a detailed investigation in the following. The main aim of this study is centered around the accurate quantification of the proportion of water volume within a uniform mixture of air, oil, and water. Some kinds of sensors, such as concave, ring, and helix designs, are found to be highly popular. The simulated TRFLC sensor is equipped with two electrodes for VCC and GND, and Fig 2 presents a 3D view of this sensor. The liquid phase, pipe, VCC electrode, and GND electrode are identifiable with violet, grey, red, and black colors, in their respective order. In Fig 3, different parts of the TRFLC sensor’s sizes are labeled. The lengths of both electrodes and the pipe are equal to L2 + D1 + D2 = 12 cm and L1 = 18 cm, respectively. Moreover, the gap between the electrodes is set to D2 = 0.5 cm, while D1 is equal to 1 cm. Additionally, regarding the remaining sizes, the internal radius, the external radius, and the outermost radius of the electrodes are defined as R1 = 2.6 cm, R2 = 3.2 cm, and R3 = 3.3 cm, respectively. For the electrodes, a copper plate, characterized by its high conductivity and, a thickness of 0.1 cm, was employed. As previously mentioned, the desired homogeneous liquid is a mixture of gas, oil, and water, and a certain percentage of each of them is considered at every stage of the simulation. Fig 4 visually presents the considered amount of each component with an orange square. For simulation, at the first step, the geometry of the sensor had to be created. So, a vacuum area was created to isolate the whole sensor from its surroundings and after that, the main pipe was made in the software. In the next, electrodes were cut based on the measurements and added to the surface of the pipe. In the next step, every part’s material was chosen. Finally. the required setting for the simulation was performed. Moreover, Fig 5A to 5e displays some plots, which belong to multislice, volume, slice, isosurface, and mesh, in their respective order. Upon simulating, a total of 231 distinct data points were extracted. Out of these, 70 data were considered as test data for the proposed network.

The simulations were done with a computing system with a core i7 (4510u) CPU. This processor provides a 4M Cache and 3.10 GHz clock speed. Besides, the utilized system had a GPU (Geforce 840m). As it was said, data collecting was done with COMSOL Multiphysics and then for processing, cleaning, and preparing data for analysis after extraction, some steps were done. First, inspecting the extracted data to Identify missing values, outliers, duplicate values and inconsistencies was performed and none of them occurred. Due to the same scale of data, there was no need for normalization and conversion. In the following, by data splitting, all of them were divided randomly for train and test categories. Finally, the generated and checked data were saved in Excel software. Statistical analysis is a methodical procedure that encompasses the selection of suitable methodologies, the utilization of pertinent software uses, and adherence to defined criteria to facilitate the efficient collection and examination of data. Within this research endeavor, the statistical approach of data splitting was employed, wherein data was randomly partitioned into train and test sets. Additionally, the software tool Excel, which offers fundamental statistical functionalities and is beneficial for straightforward analyses, was utilized. Furthermore, data quality and sampling methods served as the underpinning criteria for both data collection and analysis. Initially, the generated data underwent validation through an experimental study, after which all data points were randomly allocated. For training and testing the proposed model the MATLAB software was under run for more than 28 hours and lots of networks with a wide variety of characteristics were investigated. Finally, the best one was selected. It is to be noted that, Figs 2, 3 and 5 were obtained by the COMSOL software.

It is to be noted that, capacitance-based sensors come with several potential limitations. To begin with, the method presupposes a well-mixed and evenly distributed arrangement of phases within the measurement area. In real-world scenarios, however, flow patterns can be intricate, and phases may not exhibit uniform distribution, leading to potential inaccuracies. Additionally, the accuracy of capacitance sensors can be influenced by their geometry and size; irregular sensor shapes or inappropriate sizing for the specific application can introduce measurement errors. Furthermore, the precision of capacitance-based techniques can depend on the flow regime. Different flow patterns, such as bubbly flow, slug flow, or annular flow, may impact capacitance measurements differently. Lastly, the accuracy of capacitance-based measurements may also be contingent on the chosen measurement frequency, potentially affecting the method’s sensitivity to changes in phase. In comparison with some traditional methods, it has to be said that the efficiency of capacitance-based sensors can be notably advantageous when it comes to continuous monitoring of liquid-liquid or solid-liquid mixtures owing to their real-time data acquisition capabilities. In contrast, the efficiency of various traditional methods can exhibit significant variability based on the specific approach chosen. For instance, manual techniques like visual inspection or gravimetric analysis tend to be labor-intensive and time-consuming. On the other hand, capacitance-based sensors can achieve a high level of precision in specific applications, such as monitoring liquid levels in pipes or quantifying concentration in process industries with a minimal maintenance cost.

## 3. Development of the predicting system

**Fig 6:**
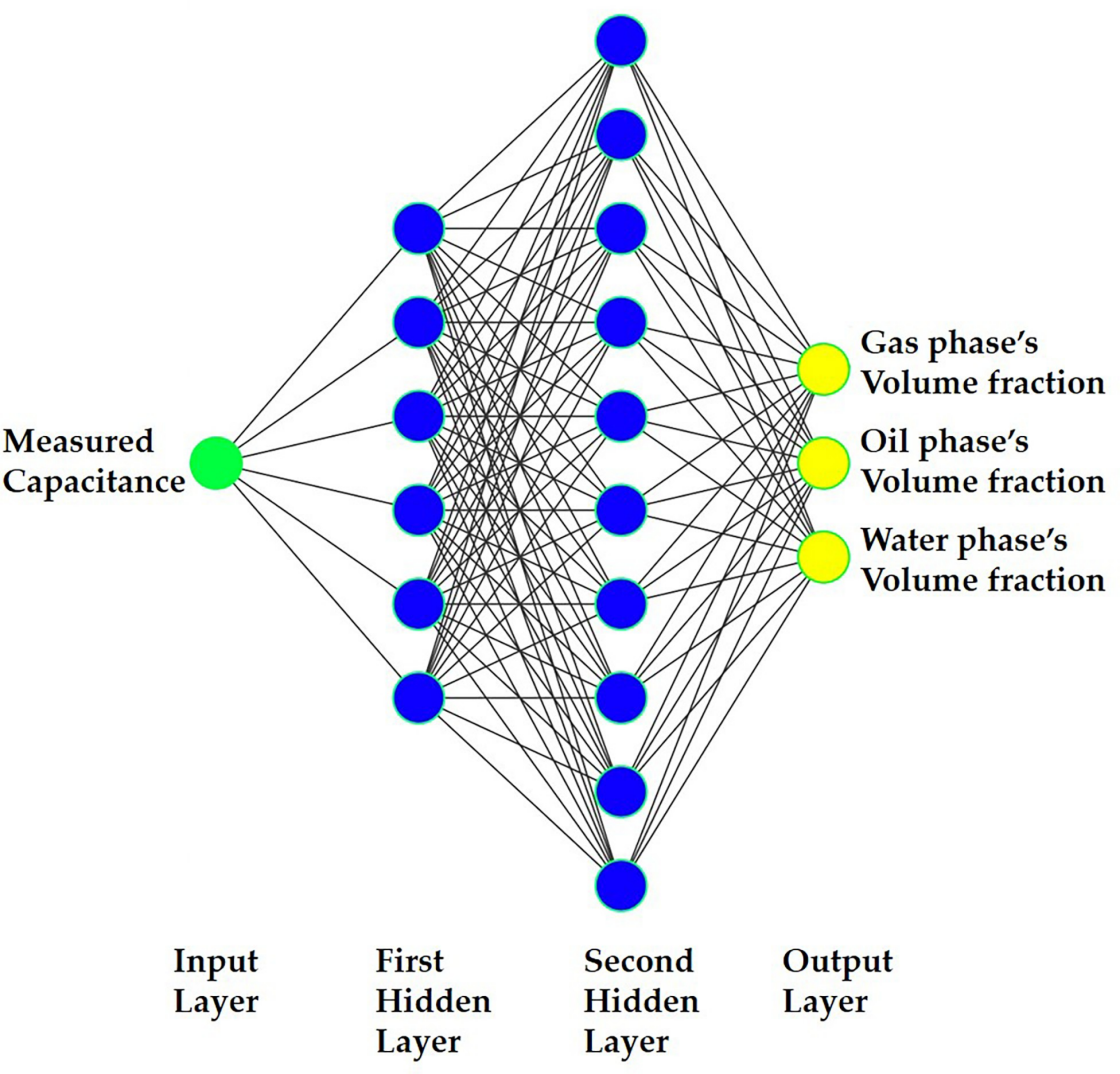
The proposed networks’ different layers [20].

Network-based models have been successful in engineering applications for prediction. These models differ in their node functionality. The Multilayer Perceptron (MLP) consists of layers: input, output, and hidden layers, each with neurons and activation functions (e.g., sigmoid or linear). During training, weights and biases are adjusted iteratively to minimize RMSE. In contrast, the Radial Basis Function (RBF) network uses linear parameters for prediction. It employs Gaussian basis functions with mean and standard deviation for input processing. The output layer applies a linear function. The number of hidden layer nodes can match data instances, but for large datasets, a smaller, carefully selected set of data points is preferable. Nowadays, among neural networks, MLP continues to hold the position of being both the most favored and widely utilized. Hence, a number of investigations, particularly measuring, can be found that have utilized the mentioned kind of network [24–29]. The domain of Artificial Intelligence (AI) is teeming with a wide range of utilizations spanning various divisions, including retail, finance, transportation, and particularly, education [40]. At the heart of AI lies the Artificial Neural Network (ANN), an intricately sophisticated mathematical method that utilizes neurons arranged in singular or multiple layers to carry out powerful computations. Within the ANN skeleton, there exist diverse type of helpful models, each possessing unique characteristics. One such a network is the multilayer perceptron (MLP), renowned for its unwavering exactness and remarkable ability to accurately rough estimates [41]. The MLP model consists of two crucial subsets of data: the train set and the test set. The train set comprises carefully curated data points used to train the model’s cognitive abilities, while the test set contains unfamiliar data to evaluate the network’s effectiveness and accuracy [42]. To achieve an optimal configuration for the ANN with low Mean Absolute Error (MAE), a comprehensive array of structures was thoroughly assessed, oscillating between a spectrum of framework properties, encompassing epoch quantities, hidden layers, and activation functions. Eventually, the most effective arrangement was presented as a state-of-the-art and optimum estimating technique. In this study, the MLP model is designed with a 1-input configuration, incorporating capacities obtained from a TRFLC sensor. A comprehensive suite of 231 simulations was meticulously orchestrated using COMSOL Multiphysics software, covering various combinations of three phases, with gradual adjustments made to the phases’ portions, extending from 0 to 100 percent with intervals of 5 percent. A random selection process was employed to choose 161 cases, which represent 70% of the total emulations available in this extensive collection, for use as the train data. Meanwhile, 70 instances, amounting to 30% of the entire dataset, were reserved as test data for evaluation purposes. After conducting exhaustive experimentation involving diverse network configurations comprising varying numbers of neurons and layers, the setup that exhibited the smallest MAE surfaced as the ultimate network. The number of neurons in the input layer, hidden layer 1, hidden layer 2, and output layer were equal to 1, 6, 10, and 3 neurons, respectively. Also, the activation function of neurons in hidden layer 1, hidden layer 2, and output layer are Tansig, Tansig, and Purelin, in their order. Besides, Levenberg-Marquardt [43, 44] was considered as the method of learning, and 400 epochs were utilized. Furthermore, the architecture of the proposed network is visually depicted in Fig 6. Also, Eqs (4) and (5) determine the MAE and the mean relative error percentage (MRE%) for an MLP model, with N denoting the count of observations, and X (Sim) and X (Pred) representing the simulated (COMSOL Multiphysics) and predicted (MLP) values, respectively [30].


$$MAE=\frac{1}{N}\sum_{i=1}^{z}\left|x_{i}(Sim)-x_{i}(Pred)\right|$$ (4)

$$MRE\%=100\times \frac{1}{N}\sum_{i=1}^{N}\left|\frac{x_{i}(Sim)-x_{i}(Pred)}{x_{i}(Sim)}\right|$$ (5)

## 4. Results and discussion

**Fig 7:**
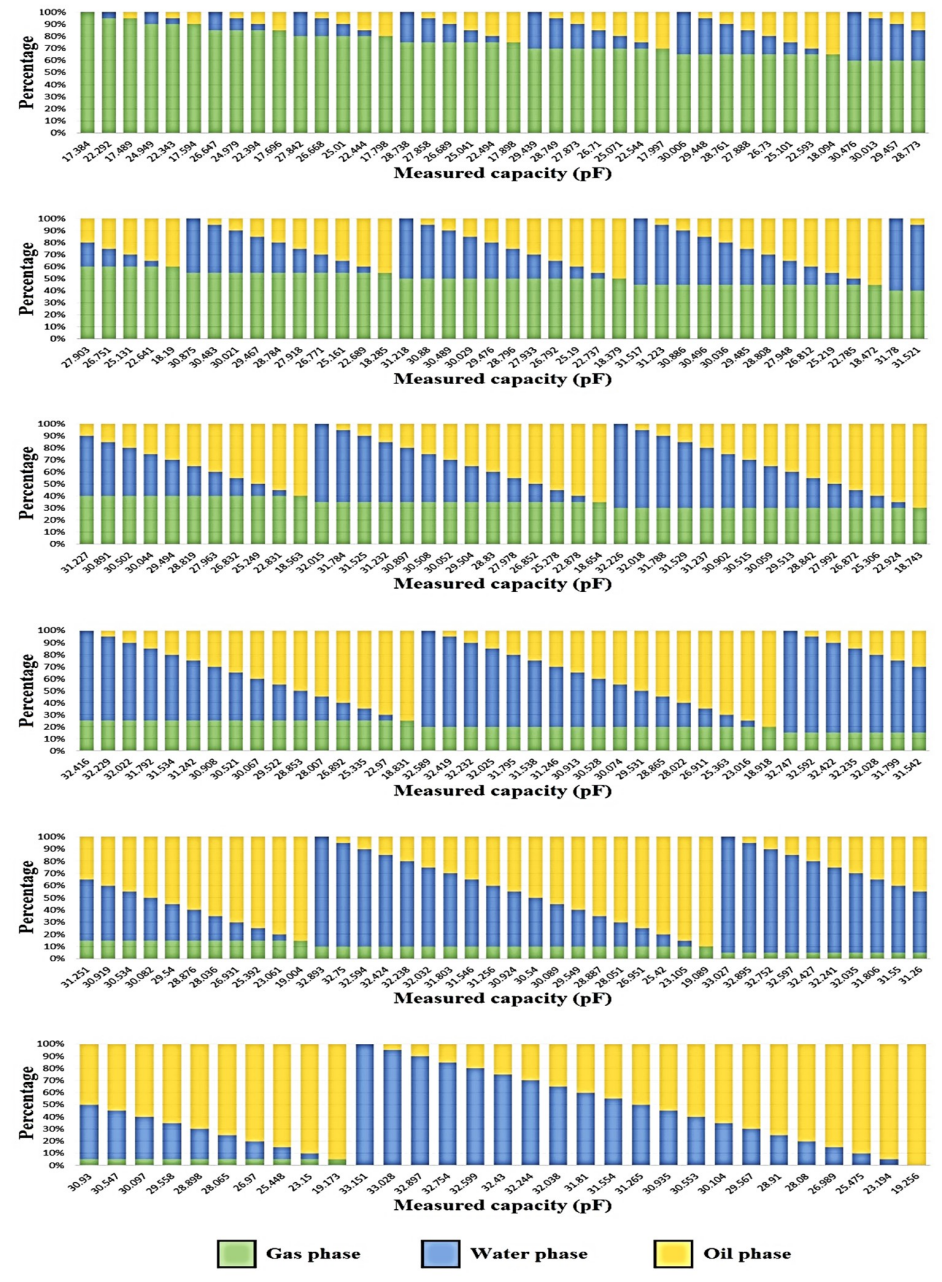
Measured capacity of different combinations of the three-phase gas-oil-water fluid.

**Fig 8:**
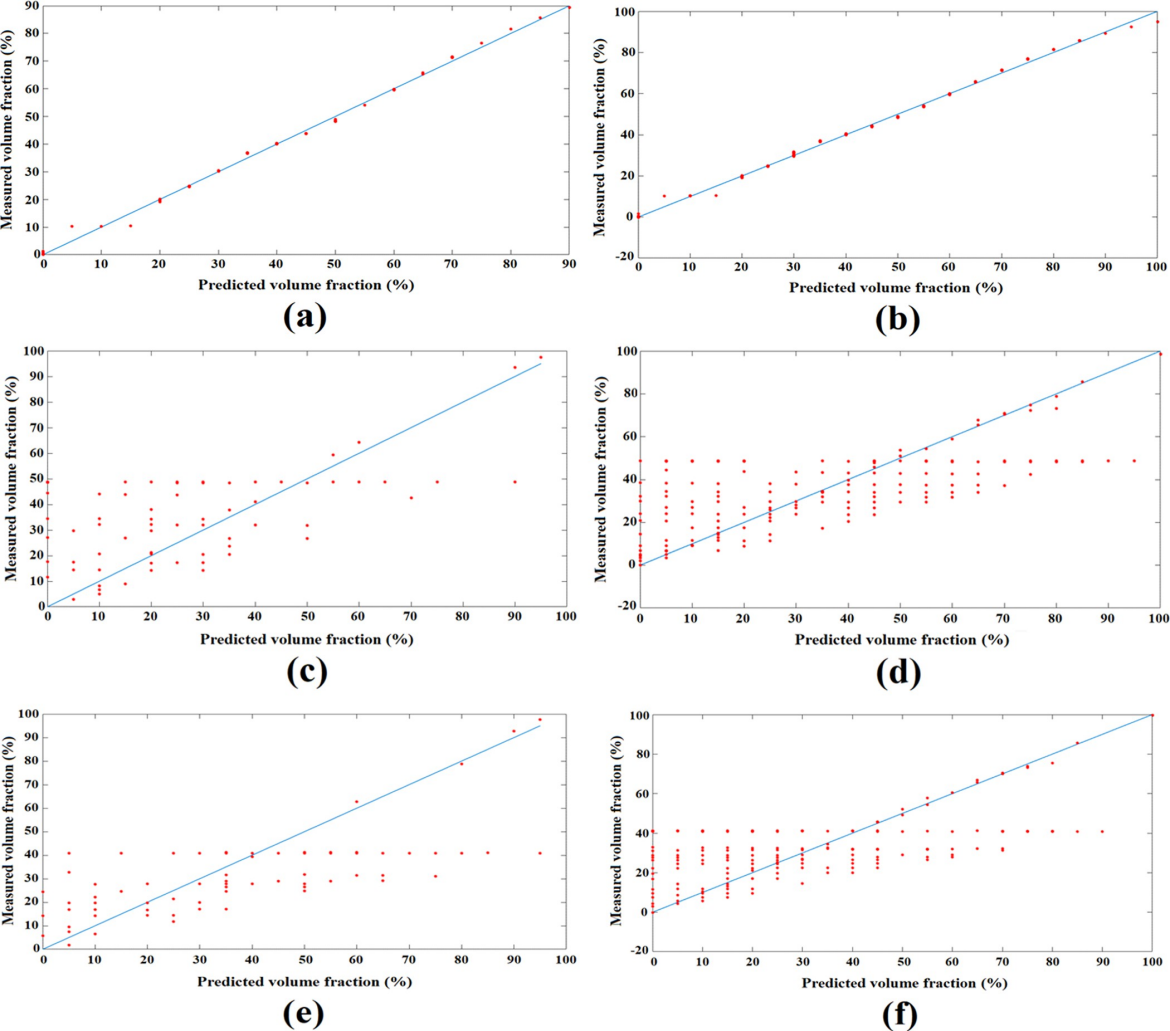
Results obtained from intelligent measuring utilizing the proposed network, (a) water phase’s test data, (b) water phase’s train data, (c) gas phase’s test data, (d) gas phase’s train data, (e) oil phase’s test data, and (f) oil phase’s train data.

As previously stated, a total of 231 datasets have been obtained through simulations utilizing the powerful COMSOL Multiphysics software. These datasets were then partitioned into two distinct groups for training and testing. Specifically, 70% of the data, equivalent to 161 cases, was allocated for the training phase, while the remaining 30%, consisting of 70 cases, was reserved for testing. All outcomes obtained from simulations are presented in Fig 7. This figure was created by the Excel software. It is important to note that the division of data between the training and testing sets was performed randomly, ensuring unbiased and robust evaluations. All 70 test cases can be seen in Table 1. In the quest to identify the most optimal architecture, numerous networks were subjected to experimentation, with the finest candidate eventually being selected. The results derived from the presented ANN are influenced by two significant factors, namely real data and predicted data. The former is generated through a simulation process, while the latter is provided by the proposed neural network. Both real and predicted data about each phase can be observed in Fig 8. Specifically, Fig 8A and 8B depict the proximity of real and predicted data concerning the water phase. These figures serve as regression diagrams, visually representing the relationship between the train and test data for the water phase, respectively. Regression serves as a statistical tool to assess the strength of association between two variables, quantifying their interdependency. In the context of this study, the MAE has been computed for both the train and test data, yielding values of 1.43 and 1.66, respectively. The proposed MLP model, fortified by a precise prediction system, which demonstrates its competence in accurately gauging the water volume fraction, achieving this feat with a remarkably low Mean Absolute Error. This pivotal accomplishment is attributed to the fusion of an ANN with an optimized capacitive sensor, which enhances the predictive capabilities of the model. Evaluating the model’s performance through Fig 8A and 8B by the MATLAB software, reveals a favorable outcome, as neither underfitting nor overfitting is observed. The integrity of the presented model remains intact, instilling trustworthiness in its predictive capabilities. Besides, it is to be noted that the reported error means that the proposed measurement system can measure the volume of water in a homogeneous gas-oil-water liquid, precisely. If the presented system wants to be used in industries, like any other system, its error rate, which is a very favorable number, should be considered and included in their desired calculations.

**Table 1:** Heat map of the proposed network’s test data.

Gas	Water	Oil	Relative permittivity	Measured capacity (pF)
Volume fraction (%)	Volume fraction (%)	Volume fraction (%)		
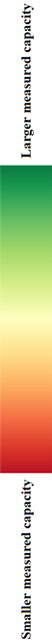	10	15	75	13.9	26.951
50	40	10	33.12	30.489
0	65	35	53.42	32.038
35	20	45	17.54	27.978
40	60	0	49	31.78
15	5	80	5.96	23.061
15	70	15	57.18	32.235
0	55	45	45.54	31.554
35	30	35	25.42	29.504
60	15	25	13.3	26.751
5	10	85	10.02	25.448
50	15	35	13.42	26.792
10	65	25	53.3	32.032
60	10	30	9.36	25.131
55	35	10	29.12	30.021
30	65	5	53.06	32.018
0	25	75	21.9	28.91
20	65	15	53.18	32.025
60	5	35	5.42	22.641
30	60	10	49.12	31.788
10	0	90	2.08	19.089
25	70	5	57.06	32.229
5	60	35	49.42	31.806
25	50	25	41.3	31.242
90	10	0	9	24.949
65	0	35	1.42	18.094
15	75	10	61.12	32.422
65	15	20	13.24	26.73
25	40	35	33.42	30.521
50	30	20	25.24	29.476
0	80	20	65.24	32.599
35	25	40	21.48	28.83
25	60	15	49.18	31.792
0	15	85	14.02	26.989
20	0	80	1.96	18.918
65	20	15	17.18	27.888
20	10	70	9.84	25.363
25	75	0	61	32.416
85	5	10	5.12	22.394
80	5	15	5.18	22.444
15	40	45	33.54	30.534
60	30	10	25.12	29.457
15	85	0	69	32.747
45	10	45	9.54	25.219
35	35	30	29.36	30.052
55	10	35	9.42	25.161
45	55	0	45	31.517
15	25	60	21.72	28.876
40	50	10	41.12	31.227
20	70	10	57.12	32.232
20	20	60	17.72	28.022
0	70	30	57.36	32.244
55	15	30	13.36	26.771
30	70	0	57	32.226
5	35	60	29.72	30.097
25	5	70	5.84	22.97
60	0	40	1.48	18.19
30	50	20	41.24	31.237
75	5	20	5.24	22.494
0	60	40	49.48	31.81
30	20	50	17.6	27.992
40	55	5	45.06	31.521
25	10	65	9.78	25.335
70	25	5	21.06	28.749
55	30	15	25.18	29.467
80	10	10	9.12	25.01
45	40	15	33.18	30.496
40	25	35	21.42	28.819
10	35	55	29.66	30.089
20	75	5	61.06	32.419

As it is clear from Fig 8, the presented metering system can predict the water volume fraction, accurately. It is because of a basic concept in the physics world. Two imperative characteristics in flow measurement are relative permittivity and density. In capacitance-based sensors, the relative permittivity (also known as the dielectric constant) plays a crucial role in determining the capacitance of the sensor. The relative permittivity of water, oil, and gas is equal to 81, 2.2, and 1, respectively [25]. Due to the vast difference between their ε_r_ and based on Eq (3) it is clear that the measured capacity of water is much higher than two other phases, this can be seen in Fig 7 and Table 1. Hence, the proposed network could predict the volume fraction of the water phase very accurately, because through the capacitance-based method the obtained capacity for any percentage of the water phase was much higher and more predictable. On the other hand, the provided network has only one input, which is the generated capacity. As it is clear in Table 2, due to the very close relative permittivity of gas and oil phases, in two completely different cases, two almost identical capacitances have been obtained, which makes the network unable to detect the volume of oil and gas phases. All in all, based on the very close relative permittivity of gas and oil, the used capacitance-based sensor, which highly depends on ε_r_was unable to predict these phases. So, the limitation of the proposed system is the relative permittivity of phases, the more difference between them, the better results can be achieved through this method. In addition, in different temperature conditions, different liquids will have different relative permittivity, which will strongly affect the results of the capacitance-based method. The studies performed in this article were conducted at room temperature (about 300 degrees Kelvin), which can be another limitation of the proposed metering system. It is suggested to consider the temperature changes of each phase and present another metering system independent of the mentioned parameter for future works. So, it can be realized that capacitance-based techniques are predominantly tailored for measuring two-phase flows, such as gas-liquid or solid-liquid systems. Their effectiveness might diminish when applied to the more intricate scenarios of multiphase flows involving three or more phases.

**Table 2:** An example to show the proximity of some data obtained from the capacitance-based sensor.

Gas	Water	Oil	Relative permittivity	Measured capacity (pF)
Volume fraction (%)	Volume fraction (%)	Volume fraction (%)		
5	35	60	9.9067	25.395
25	35	40	9.8267	25.357

Moreover, it can be said that the attenuation coefficient for the gamma-ray method at a given photon energy is highly dependent on the density of the absorber [45]. For a water-oil-gas fluid, the density of water, oil, and gas is equal to 1, 0.9, and 0.001 gr/cm^3^, respectively. Therefore, according to Eq (1), the counting of received photons highly depends on the density of the matter. This way, the mentioned method utilizing a Cesium source, cannot recognize water and oil phases accurately and can be utilized to predict the volume fraction of liquid and gas phases. Also, because the linear attenuation coefficients of energy in oil and water are relatively close, slight inaccuracies in intensity measurements can be compounded into huge errors in thickness calculation. Besides, in some cases, due to a lower energy level of the gamma-ray in the Americium (Am) source, oil and water phases’ volumes can be predicted because sensitivity relies on the atomic number rather than the density. For example, in [46] utilizing the Am source the volume fraction of water was investigated and results showed good accuracy, but its accuracy is lower than the obtained error in the presented model. All in all, conversely to the capacitance-based sensors, in gamma-ray ones, water and oil have a close density and this leads to too close counted photon numbers. This way, the ANN cannot predict their volumes. Hence, a combined method using both sensors to accurately measure all 3 phases is suggested for future works. For measuring volume fraction utilizing the gamma-ray attenuation, there is a need for a photon source, one or two detectors, and a tube shield with an output window. After the photon exits from the output window, it passes through the tube containing the desired liquid and according to the number of photons received in the detector, the volume fraction of water will be obtained. One of the challenges of this method is shielding the structure because it can have effects on human’s body. Therefore utilizing capacitance-based and gamma-ray sensors the volume fraction of water and gas can be measured, respectively. Besides, a kind of mixed method, which can predict volume fractions of all three phases, can be considered as a novel work that can be published in future studies. All in all, when relative permittivity of oil and gas is so close, the capacitance-based sensors’ data helps the network to estimate the volume fraction of water. In the case of density which gas phase’s density is so different from others, the gamma-ray attenuation can help the model to predict void fraction. Finally, by subtracting both of these volumes from 100, the volume fraction of oil is predicted.

Data utilized in this study were generated through Finite Element Method (FEM) simulations, which provided precise and validated inputs for the presented ANN model. This ensured that the calibration points used in real-world applications are devoid of noise and uncertainties, thus serving as reliable and accurate training data for the ANN to predict volume fraction, they can be considered as valid and corrected values. The robustness of the ANN, combined with the precision of the FEM-generated data, supports the interpretation of the trends in our study. Using of an accurate measurement system in industries such as oil is very vital, because these industries, as the sellers of their products, must be aware of the level of dilution of liquids for financial calculations. In this way, the financial loss of sellers and buyers of such products will be prevented. The reported MAE for the training set was equal to 1.43 which is low and shows the precision of the presented network. Simultaneously, the low value of MAE related to the testing set was 1.66 which illustrates the correction of the proposed model. In fact, the presented metering system is both accurate and precise and from obtained results it can be concluded that both overfitting and underfitting have not occurred.

## 5. Conclusion

There are lots of industries where precise measuring is a must. For example, in oil sectors that sell products, utilizing an accurate metering system like the proposed one can be a key step toward calculating financial-related things, correctly. The study showcased a novel metering system for quantifying the water volume fraction in a three-phase homogeneous regime. The presented model can predict the mentioned void fraction with a low MAE equal to 1.6 which demonstrated its precision and accuracy. To attain the previously mentioned objective an MLP ANN was devised. The model contained 1 input, the measured capacity of the optimized sensor utilizing the COMSOL Multiphysics software, and 3 outputs, related to the volume fraction of water, oil, and gas phases. The produced data were divided randomly between two groups, train, and test. Among all 231 data, 161 of them were considered to train the proposed model and the rest of them were taken for testing it. Based on the obtained results, it was taken that due to the reliance on relative permittivity, capacitance-based sensors can predict only the water volume fraction. Due to the very close relative permittivity of gas and oil, the used capacitance-based sensor, which highly depends on the mentioned physical parameter, was unable to predict these phases. To measure two other phases, oil and gas, another method called gamma-ray attenuation was suggested. This method depends on density and due to the close densities of water and oil phases, can predict gas volume fraction, precisely. So, a combined method using both sensors to accurately measure all 3 phases was suggested for future works.

## Supporting information

S1 File (DOCX)
