# Supplementary material for: Measuring volume fractions of a three-phase flow without separation utilizing an approach based on artificial intelligence and capacitive sensors
Source: PLoS One. 2024 May 16;19(5):e0301437. doi: 10.1371/journal.pone.0301437 (PMC11098402; doi:10.1371/journal.pone.0301437)
Supplement: S1 File — (DOCX) [file pone.0301437.s001.docx]

**Supplementary data**

Measuring Volume Fractions of a Three-Phase Flow without Separation Utilizing an Approach Based on Artificial Intelligence and Capacitive Sensors

Abdulilah Mohammad Mayet ^1^, Farhad Fouladinia ^2^, Seyed Mehdi Alizadeh^3^, Hala H. Alhashim ^4^, John William Grimaldo Guerrero ^5,^, Hassen Loukil ^1^, Muneer Parayangat ^1^, Ehsan Nazemi ^6,^ * and Neeraj Kumar Shukla ^1^

^1^ Electrical Engineering Department, King Khalid University, Abha 61411, Saudi Arabia; amayet@kku.edu.sa; hloukil@kku.edu.sa; mparayangat@kku.edu.sa; nshukla@kku.edu.sa

^2^ Faculty of engineering, Rzeszow University of Technology, Powstancow Warszawy 12, 35-959 Rzeszow, Poland; farhad.fouladi93@gmail.com

^3^ Petroleum Engineering Department, Australian University, West Mishref 13015, Kuwait; s.alizadeh@au.edu.kw

^4^ Department of Physics, College of Science, Imam Abdulrahman Bin Faisal University, Dammam 31441, Saudi Arabia; halhashim@iau.edu.sa

^5^ Department of Energy, Universidad de la Costa, Barranquilla 080001, Colombia; jgrimald1@cuc.edu.co

^6^ Faculty of engineering, University of Southampton, UK; e.nazemi@soton.ac.uk

**Table 1. Measured capacities which were obtained by COMSOL Multiphysics software**

| **Maxwell capacitance (pF)** | **Gas VF (%)** | **Water VF (%)** | **Oil VF (%)** | |
| --- | --- | --- | --- | --- |
| 17.384 | 100 | 0 | 0 |  |
| 22.292 | 95 | 5 | 0 |  |
| 17.489 | 95 | 0 | 5 |  |
| 24.949 | 90 | 10 | 0 |  |
| 22.343 | 90 | 5 | 5 |  |
| 17.594 | 90 | 0 | 10 |  |
| 26.647 | 85 | 15 | 0 |  |
| 24.979 | 85 | 10 | 5 |  |
| 22.394 | 85 | 5 | 10 |  |
| 17.696 | 85 | 0 | 15 |  |
| 27.842 | 80 | 20 | 0 |  |
| 26.668 | 80 | 15 | 5 |  |
| 25.01 | 80 | 10 | 10 |  |
| 22.444 | 80 | 5 | 15 |  |
| 17.798 | 80 | 0 | 20 |  |
| 28.738 | 75 | 25 | 0 |  |
| 27.858 | 75 | 20 | 5 |  |
| 26.689 | 75 | 15 | 10 |  |
| 25.041 | 75 | 10 | 15 |  |
| 22.494 | 75 | 5 | 20 |  |
| 17.898 | 75 | 0 | 25 |  |
| 29.439 | 70 | 30 | 0 |  |
| 28.749 | 70 | 25 | 5 |  |
| 27.873 | 70 | 20 | 10 |  |
| 26.71 | 70 | 15 | 15 |  |
| 25.071 | 70 | 10 | 20 |  |
| 22.544 | 70 | 5 | 25 |  |
| 17.997 | 70 | 0 | 30 |  |
| 30.006 | 65 | 35 | 0 |  |
| 29.448 | 65 | 30 | 5 |  |
| 28.761 | 65 | 25 | 10 |  |
| 27.888 | 65 | 20 | 15 |  |
| 26.73 | 65 | 15 | 20 |  |
| 25.101 | 65 | 10 | 25 |  |
| 22.593 | 65 | 5 | 30 |  |
| 18.094 | 65 | 0 | 35 |  |
| 30.476 | 60 | 40 | 0 |  |
| 30.013 | 60 | 35 | 5 |  |
| 29.457 | 60 | 30 | 10 |  |
| 28.773 | 60 | 25 | 15 |  |
| 27.903 | 60 | 20 | 20 |  |
| 26.751 | 60 | 15 | 25 |  |
| 25.131 | 60 | 10 | 30 |  |
| 22.641 | 60 | 5 | 35 |  |
| 18.19 | 60 | 0 | 40 |  |
| 30.875 | 55 | 45 | 0 |  |
| 30.483 | 55 | 40 | 5 |  |
| 30.021 | 55 | 35 | 10 |  |
| 29.467 | 55 | 30 | 15 |  |
| 28.784 | 55 | 25 | 20 |  |
| 27.918 | 55 | 20 | 25 |  |
| 26.771 | 55 | 15 | 30 |  |
| 25.161 | 55 | 10 | 35 |  |
| 22.689 | 55 | 5 | 40 |  |
| 18.285 | 55 | 0 | 45 |  |
| 31.218 | 50 | 50 | 0 |  |
| 30.88 | 50 | 45 | 5 |  |
| 30.489 | 50 | 40 | 10 |  |
| 30.029 | 50 | 35 | 15 |  |
| 29.476 | 50 | 30 | 20 |  |
| 28.796 | 50 | 25 | 25 |  |
| 27.933 | 50 | 20 | 30 |  |
| 26.792 | 50 | 15 | 35 |  |
| 25.19 | 50 | 10 | 40 |  |
| 22.737 | 50 | 5 | 45 |  |
| 18.379 | 50 | 0 | 50 |  |
| 31.517 | 45 | 55 | 0 |  |
| 31.223 | 45 | 50 | 5 |  |
| 30.886 | 45 | 45 | 10 |  |
| 30.496 | 45 | 40 | 15 |  |
| 30.036 | 45 | 35 | 20 |  |
| 29.485 | 45 | 30 | 25 |  |
| 28.808 | 45 | 25 | 30 |  |
| 27.948 | 45 | 20 | 35 |  |
| 26.812 | 45 | 15 | 40 |  |
| 25.219 | 45 | 10 | 45 |  |
| 22.785 | 45 | 5 | 50 |  |
| 18.472 | 45 | 0 | 55 |  |
| 31.78 | 40 | 60 | 0 |  |
| 31.521 | 40 | 55 | 5 |  |
| 31.227 | 40 | 50 | 10 |  |
| 30.891 | 40 | 45 | 15 |  |
| 30.502 | 40 | 40 | 20 |  |
| 30.044 | 40 | 35 | 25 |  |
| 29.494 | 40 | 30 | 30 |  |
| 28.819 | 40 | 25 | 35 |  |
| 27.963 | 40 | 20 | 40 |  |
| 26.832 | 40 | 15 | 45 |  |
| 25.249 | 40 | 10 | 50 |  |
| 22.831 | 40 | 5 | 55 |  |
| 18.563 | 40 | 0 | 60 |  |
| 32.015 | 35 | 65 | 0 |  |
| 31.784 | 35 | 60 | 5 |  |
| 31.525 | 35 | 55 | 10 |  |
| 31.232 | 35 | 50 | 15 |  |
| 30.897 | 35 | 45 | 20 |  |
| 30.508 | 35 | 40 | 25 |  |
| 30.052 | 35 | 35 | 30 |  |
| 29.504 | 35 | 30 | 35 |  |
| 28.83 | 35 | 25 | 40 |  |
| 27.978 | 35 | 20 | 45 |  |
| 26.852 | 35 | 15 | 50 |  |
| 25.278 | 35 | 10 | 55 |  |
| 22.878 | 35 | 5 | 60 |  |
| 18.654 | 35 | 0 | 65 |  |
| 32.226 | 30 | 70 | 0 |  |
| 32.018 | 30 | 65 | 5 |  |
| 31.788 | 30 | 60 | 10 |  |
| 31.529 | 30 | 55 | 15 |  |
| 31.237 | 30 | 50 | 20 |  |
| 30.902 | 30 | 45 | 25 |  |
| 30.515 | 30 | 40 | 30 |  |
| 30.059 | 30 | 35 | 35 |  |
| 29.513 | 30 | 30 | 40 |  |
| 28.842 | 30 | 25 | 45 |  |
| 27.992 | 30 | 20 | 50 |  |
| 26.872 | 30 | 15 | 55 |  |
| 25.306 | 30 | 10 | 60 |  |
| 22.924 | 30 | 5 | 65 |  |
| 18.743 | 30 | 0 | 70 |  |
| 32.416 | 25 | 75 | 0 |  |
| 32.229 | 25 | 70 | 5 |  |
| 32.022 | 25 | 65 | 10 |  |
| 31.792 | 25 | 60 | 15 |  |
| 31.534 | 25 | 55 | 20 |  |
| 31.242 | 25 | 50 | 25 |  |
| 30.908 | 25 | 45 | 30 |  |
| 30.521 | 25 | 40 | 35 |  |
| 30.067 | 25 | 35 | 40 |  |
| 29.522 | 25 | 30 | 45 |  |
| 28.853 | 25 | 25 | 50 |  |
| 28.007 | 25 | 20 | 55 |  |
| 26.892 | 25 | 15 | 60 |  |
| 25.335 | 25 | 10 | 65 |  |
| 22.97 | 25 | 5 | 70 |  |
| 18.831 | 25 | 0 | 75 |  |
| 32.589 | 20 | 80 | 0 |  |
| 32.419 | 20 | 75 | 5 |  |
| 32.232 | 20 | 70 | 10 |  |
| 32.025 | 20 | 65 | 15 |  |
| 31.795 | 20 | 60 | 20 |  |
| 31.538 | 20 | 55 | 25 |  |
| 31.246 | 20 | 50 | 30 |  |
| 30.913 | 20 | 45 | 35 |  |
| 30.528 | 20 | 40 | 40 |  |
| 30.074 | 20 | 35 | 45 |  |
| 29.531 | 20 | 30 | 50 |  |
| 28.865 | 20 | 25 | 55 |  |
| 28.022 | 20 | 20 | 60 |  |
| 26.911 | 20 | 15 | 65 |  |
| 25.363 | 20 | 10 | 70 |  |
| 23.016 | 20 | 5 | 75 |  |
| 18.918 | 20 | 0 | 80 |  |
| 32.747 | 15 | 85 | 0 |  |
| 32.592 | 15 | 80 | 5 |  |
| 32.422 | 15 | 75 | 10 |  |
| 32.235 | 15 | 70 | 15 |  |
| 32.028 | 15 | 65 | 20 |  |
| 31.799 | 15 | 60 | 25 |  |
| 31.542 | 15 | 55 | 30 |  |
| 31.251 | 15 | 50 | 35 |  |
| 30.919 | 15 | 45 | 40 |  |
| 30.534 | 15 | 40 | 45 |  |
| 30.082 | 15 | 35 | 50 |  |
| 29.54 | 15 | 30 | 55 |  |
| 28.876 | 15 | 25 | 60 |  |
| 28.036 | 15 | 20 | 65 |  |
| 26.931 | 15 | 15 | 70 |  |
| 25.392 | 15 | 10 | 75 |  |
| 23.061 | 15 | 5 | 80 |  |
| 19.004 | 15 | 0 | 85 |  |
| 32.893 | 10 | 90 | 0 |  |
| 32.75 | 10 | 85 | 5 |  |
| 32.594 | 10 | 80 | 10 |  |
| 32.424 | 10 | 75 | 15 |  |
| 32.238 | 10 | 70 | 20 |  |
| 32.032 | 10 | 65 | 25 |  |
| 31.803 | 10 | 60 | 30 |  |
| 31.546 | 10 | 55 | 35 |  |
| 31.256 | 10 | 50 | 40 |  |
| 30.924 | 10 | 45 | 45 |  |
| 30.54 | 10 | 40 | 50 |  |
| 30.089 | 10 | 35 | 55 |  |
| 29.549 | 10 | 30 | 60 |  |
| 28.887 | 10 | 25 | 65 |  |
| 28.051 | 10 | 20 | 70 |  |
| 26.951 | 10 | 15 | 75 |  |
| 25.42 | 10 | 10 | 80 |  |
| 23.105 | 10 | 5 | 85 |  |
| 19.089 | 10 | 0 | 90 |  |
| 33.027 | 5 | 95 | 0 |  |
| 32.895 | 5 | 90 | 5 |  |
| 32.752 | 5 | 85 | 10 |  |
| 32.597 | 5 | 80 | 15 |  |
| 32.427 | 5 | 75 | 20 |  |
| 32.241 | 5 | 70 | 25 |  |
| 32.035 | 5 | 65 | 30 |  |
| 31.806 | 5 | 60 | 35 |  |
| 31.55 | 5 | 55 | 40 |  |
| 31.26 | 5 | 50 | 45 |  |
| 30.93 | 5 | 45 | 50 |  |
| 30.547 | 5 | 40 | 55 |  |
| 30.097 | 5 | 35 | 60 |  |
| 29.558 | 5 | 30 | 65 |  |
| 28.898 | 5 | 25 | 70 |  |
| 28.065 | 5 | 20 | 75 |  |
| 26.97 | 5 | 15 | 80 |  |
| 25.448 | 5 | 10 | 85 |  |
| 23.15 | 5 | 5 | 90 |  |
| 19.173 | 5 | 0 | 95 |  |
| 33.151 | 0 | 100 | 0 |  |
| 33.028 | 0 | 95 | 5 |  |
| 32.897 | 0 | 90 | 10 |  |
| 32.754 | 0 | 85 | 15 |  |
| 32.599 | 0 | 80 | 20 |  |
| 32.43 | 0 | 75 | 25 |  |
| 32.244 | 0 | 70 | 30 |  |
| 32.038 | 0 | 65 | 35 |  |
| 31.81 | 0 | 60 | 40 |  |
| 31.554 | 0 | 55 | 45 |  |
| 31.265 | 0 | 50 | 50 |  |
| 30.935 | 0 | 45 | 55 |  |
| 30.553 | 0 | 40 | 60 |  |
| 30.104 | 0 | 35 | 65 |  |
| 29.567 | 0 | 30 | 70 |  |
| 28.91 | 0 | 25 | 75 |  |
| 28.08 | 0 | 20 | 80 |  |
| 26.989 | 0 | 15 | 85 |  |
| 25.475 | 0 | 10 | 90 |  |
| 23.194 | 0 | 5 | 95 |  |
| 19.256 | 0 | 0 | 100 |  |

**Table 2. Real volumes along with predicted ones by the proposed network.**

| Gas VF (%) | Water VF (%) | Oil VF (%) | Gas VF (%)-Predicted | Water VF (%)-Predicted | Oil VF (%)-Predicted |
| --- | --- | --- | --- | --- | --- |
| 15 | 40 | 45 | 27.72517032 | 40.36599206 | 31.91828591 |
| 0 | 5 | 95 | 40.86226629 | 10.32986492 | 48.79185525 |
| 15 | 0 | 85 | 14.41459167 | 0.378014854 | 85.5872848 |
| 20 | 50 | 30 | 24.56185994 | 48.61676009 | 26.82949214 |
| 70 | 25 | 5 | 31.18860876 | 24.51154841 | 44.30203269 |
| 5 | 10 | 85 | 40.9257236 | 10.34259373 | 48.71592618 |
| 5 | 45 | 50 | 26.30333145 | 44.25803891 | 29.4477121 |
| 85 | 0 | 15 | 85.60589173 | 0.269220038 | 14.97704205 |
| 55 | 25 | 20 | 31.63872949 | 24.60165794 | 43.76360912 |
| 35 | 10 | 55 | 40.91508216 | 10.34045627 | 48.72866197 |
| 25 | 25 | 50 | 32.35254396 | 24.74463341 | 42.90968434 |
| 30 | 20 | 50 | 26.93628871 | 19.24427617 | 53.75317183 |
| 65 | 30 | 5 | 32.1023575 | 29.55512683 | 38.35458784 |
| 75 | 20 | 5 | 73.20201041 | 20.29649766 | 6.525845853 |
| 70 | 15 | 15 | 41.12554472 | 10.38489611 | 48.47461018 |
| 0 | 95 | 5 | 4.30646755 | 92.33065953 | 3.350775467 |
| 80 | 15 | 5 | 41.10999154 | 10.38138892 | 48.49360794 |
| 85 | 10 | 5 | 40.90005999 | 10.33744212 | 48.74663745 |
| 0 | 40 | 60 | 27.67083037 | 40.52237146 | 31.81624225 |
| 15 | 80 | 5 | 9.527613841 | 81.44865761 | 9.017330528 |
| 25 | 5 | 70 | 40.86060315 | 10.32953153 | 48.79384505 |
| 0 | 25 | 75 | 32.80361801 | 24.83581674 | 42.36923852 |
| 30 | 50 | 20 | 24.62088341 | 48.47390747 | 26.91336042 |
| 40 | 30 | 30 | 31.72415288 | 30.38607154 | 37.90148907 |
| 0 | 65 | 35 | 16.87551318 | 65.90379053 | 17.22203065 |
| 35 | 60 | 5 | 19.86744554 | 59.3853686 | 20.75142777 |
| 15 | 15 | 70 | 41.24430265 | 10.41262891 | 48.32859449 |
| 25 | 50 | 25 | 24.58816638 | 48.55312567 | 26.8668376 |
| 15 | 75 | 10 | 11.79666325 | 76.69229623 | 11.50708612 |
| 0 | 45 | 55 | 26.28062889 | 44.31721752 | 29.41122642 |
| 40 | 55 | 5 | 22.45558337 | 53.56685859 | 23.98408521 |
| 50 | 35 | 15 | 28.99827039 | 36.69366306 | 34.3176026 |
| 20 | 15 | 65 | 41.23004445 | 10.4092255 | 48.34619941 |
| 50 | 20 | 30 | 52.08380893 | 19.86728123 | 28.03226667 |
| 40 | 45 | 15 | 26.47567726 | 43.80620441 | 29.72727063 |
| 55 | 10 | 35 | 40.90869553 | 10.33917446 | 48.73630454 |
| 35 | 0 | 65 | 34.41187747 | 0.084755239 | 65.50944527 |
| 10 | 45 | 45 | 26.33039014 | 44.18740386 | 29.49129982 |
| 0 | 80 | 20 | 9.435821576 | 81.64055645 | 8.917124445 |
| 30 | 30 | 40 | 31.57105392 | 30.72124484 | 37.719268 |
| 25 | 45 | 30 | 26.40157328 | 44.00104934 | 29.60650038 |
| 40 | 15 | 45 | 41.181671 | 10.39781567 | 48.40578958 |
| 25 | 60 | 15 | 19.78008126 | 59.57831855 | 20.64576053 |
| 25 | 35 | 40 | 28.89592849 | 36.9722738 | 34.14130546 |
| 35 | 20 | 45 | 32.73786155 | 19.38024038 | 47.82699035 |
| 25 | 65 | 10 | 17.07592985 | 65.47205459 | 17.45355096 |
| 30 | 55 | 15 | 22.38519328 | 53.72826913 | 23.89300666 |
| 15 | 65 | 20 | 17.00093545 | 65.63367639 | 17.36684842 |
| 10 | 80 | 10 | 9.501369815 | 81.50352624 | 8.988677478 |
| 45 | 0 | 55 | 45.599915 | -0.076445461 | 54.48031402 |
| 20 | 5 | 75 | 40.86091696 | 10.32959443 | 48.79346961 |
| 55 | 30 | 15 | 31.94536884 | 29.90078722 | 38.16576818 |
| 10 | 25 | 65 | 32.63459365 | 24.80139377 | 42.57200671 |
| 25 | 30 | 45 | 31.49953555 | 30.8777423 | 37.63422028 |
| 0 | 60 | 40 | 19.58174059 | 60.0156838 | 20.40654992 |
| 45 | 45 | 10 | 26.49717737 | 43.7495084 | 29.76247482 |
| 25 | 15 | 60 | 41.21731571 | 10.40620186 | 48.36190107 |
| 55 | 40 | 5 | 27.86603805 | 39.9578635 | 32.18555417 |
| 45 | 5 | 50 | 40.85946847 | 10.32930406 | 48.7952026 |
| 20 | 10 | 70 | 40.9201871 | 10.34148134 | 48.72255264 |
| 35 | 55 | 10 | 22.42045421 | 53.64743944 | 23.93860455 |
| 0 | 100 | 0 | 3.070428368 | 94.90463937 | 2.011484294 |
| 50 | 5 | 45 | 40.85920456 | 10.32925116 | 48.79551834 |
| 15 | 60 | 25 | 19.70323901 | 59.74787649 | 20.55297291 |
| 30 | 45 | 25 | 26.42790586 | 43.93191181 | 29.64931587 |
| 0 | 75 | 25 | 11.6887555 | 76.91913331 | 11.38804139 |
| 5 | 35 | 60 | 28.81831782 | 37.18722612 | 34.00394707 |
| 40 | 10 | 50 | 40.91343266 | 10.34012515 | 48.73063591 |
| 10 | 20 | 70 | 10.45637148 | 19.0193957 | 70.42759088 |
| 80 | 10 | 10 | 40.90142856 | 10.33771662 | 48.74499992 |
| 45 | 20 | 35 | 45.85093015 | 19.70958129 | 34.41050581 |
| 40 | 0 | 60 | 41.09529113 | 0.003705615 | 58.88792 |
| 35 | 25 | 40 | 32.13738719 | 24.70150612 | 43.16710476 |
| 5 | 30 | 65 | 31.22145699 | 31.48653076 | 37.30324389 |
| 20 | 45 | 35 | 26.37947965 | 44.05897344 | 29.57066096 |
| 0 | 85 | 15 | 7.456962765 | 85.7705294 | 6.763856604 |
| 25 | 40 | 35 | 27.76174779 | 40.26038901 | 31.98731389 |
| 45 | 25 | 30 | 31.91100564 | 24.65616971 | 43.43791406 |
| 5 | 50 | 45 | 24.46885308 | 48.84130665 | 26.69789009 |
| 15 | 85 | 0 | 7.543765765 | 85.58959917 | 6.858078583 |
| 20 | 0 | 80 | 21.07380916 | 0.246995094 | 78.8684517 |
| 0 | 10 | 90 | 40.927579 | 10.34296669 | 48.71370533 |
| 60 | 35 | 5 | 29.04298285 | 36.57369914 | 34.39286868 |
| 100 | 0 | 0 | 99.72334034 | -0.049389071 | -0.011019489 |
| 70 | 0 | 30 | 69.99579548 | -0.03972535 | 29.72187967 |
| 20 | 80 | 0 | 9.567006007 | 81.36629478 | 9.060343875 |
| 15 | 70 | 15 | 14.31438123 | 71.37559099 | 14.30873524 |
| 10 | 15 | 75 | 41.25952043 | 10.41627947 | 48.30978654 |
| 60 | 5 | 35 | 40.85871057 | 10.32915213 | 48.79610936 |
| 50 | 0 | 50 | 49.2196403 | -0.151606102 | 50.97124414 |
| 15 | 20 | 65 | 13.22153036 | 19.01537979 | 67.67105933 |
| 10 | 85 | 5 | 7.506531108 | 85.66721234 | 6.81765943 |
| 75 | 10 | 15 | 40.90283752 | 10.33799923 | 48.74331405 |
| 20 | 25 | 55 | 32.45675433 | 24.76555682 | 42.78496891 |
| 5 | 95 | 0 | 4.316995318 | 92.30874344 | 3.362175304 |
| 90 | 10 | 0 | 40.8987729 | 10.33718399 | 48.74817746 |
| 5 | 15 | 80 | 41.27494579 | 10.41999775 | 48.29070398 |
| 30 | 70 | 0 | 14.43431712 | 71.12097942 | 14.443536 |
| 15 | 25 | 60 | 32.54774276 | 24.78386364 | 42.67603896 |
| 15 | 35 | 50 | 28.85681451 | 37.08021793 | 34.07246638 |
| 0 | 0 | 100 | -0.149653691 | 1.63572141 | 98.63927166 |
| 15 | 45 | 40 | 26.35278632 | 44.12885579 | 29.527461 |
| 10 | 90 | 0 | 5.794679284 | 89.2328 | 4.962051994 |
| 70 | 30 | 0 | 32.17674962 | 29.39080013 | 38.44459185 |
| 5 | 55 | 40 | 22.19790998 | 54.1567055 | 23.65169767 |
| 40 | 60 | 0 | 19.91094462 | 59.28922779 | 20.80411002 |
| 10 | 30 | 60 | 31.28966575 | 31.33709964 | 37.38453147 |
| 80 | 20 | 0 | 75.50137564 | 20.29178588 | 4.235474745 |
| 0 | 20 | 80 | 7.491618778 | 19.13037202 | 73.27766421 |
| 25 | 0 | 75 | 25.24322024 | 0.188233016 | 74.65665593 |
| 20 | 55 | 25 | 22.30537417 | 53.91104551 | 23.78998317 |
| 65 | 15 | 20 | 41.13354142 | 10.38671257 | 48.46482918 |
| 0 | 15 | 85 | 41.29139901 | 10.42398253 | 48.27033098 |
| 40 | 50 | 10 | 24.68576636 | 48.31655023 | 27.00587729 |
| 60 | 30 | 10 | 32.02794042 | 29.71914815 | 38.26491369 |
| 45 | 30 | 25 | 31.79750754 | 30.22532305 | 37.988953 |
| 30 | 0 | 70 | 29.05132493 | 0.143812542 | 70.84319398 |
| 20 | 30 | 50 | 31.42875778 | 31.03261808 | 37.55005435 |
| 30 | 25 | 45 | 32.2522552 | 24.72452293 | 43.02968101 |
| 5 | 70 | 25 | 14.23431554 | 71.54548517 | 14.21882313 |
| 10 | 75 | 15 | 11.76968141 | 76.74902266 | 11.47731268 |
| 5 | 75 | 20 | 11.72921465 | 76.8340911 | 11.43266772 |
| 0 | 55 | 45 | 22.1618247 | 54.23908915 | 23.60536906 |
| 55 | 0 | 45 | 54.36023807 | -0.191285114 | 45.85665668 |
| 70 | 20 | 10 | 70.40422172 | 20.26710271 | 9.347750894 |
| 90 | 5 | 5 | 40.85742027 | 10.32889344 | 48.79765312 |
| 20 | 75 | 5 | 11.83714166 | 76.60718604 | 11.55176117 |
| 0 | 35 | 65 | 28.80054453 | 37.23688105 | 33.97206213 |
| 65 | 20 | 15 | 66.92554743 | 20.20821576 | 12.87865405 |
| 55 | 20 | 25 | 57.75105402 | 20.00638003 | 22.23709953 |
| 20 | 35 | 45 | 28.87759401 | 37.02277299 | 34.10913645 |
| 45 | 50 | 5 | 24.71151466 | 48.25400888 | 27.04268712 |
| 5 | 90 | 5 | 5.771678521 | 89.28068297 | 4.937144615 |
| 45 | 15 | 40 | 41.1711289 | 10.39536044 | 48.41874469 |
| 40 | 25 | 35 | 32.02683516 | 24.67936371 | 43.29935546 |
| 15 | 55 | 30 | 22.2696847 | 53.99268359 | 23.74400483 |
| 15 | 30 | 55 | 31.35878259 | 31.18576812 | 37.46681238 |
| 20 | 65 | 15 | 17.03845717 | 65.55282313 | 17.41021757 |
| 10 | 10 | 80 | 40.92384975 | 10.34221715 | 48.71816901 |
| 10 | 5 | 85 | 40.86156365 | 10.32972407 | 48.7926959 |
| 5 | 40 | 55 | 27.68810593 | 40.4727224 | 31.84861717 |
| 60 | 40 | 0 | 27.88484677 | 39.90309059 | 32.221519 |
| 25 | 55 | 20 | 22.34093174 | 53.8296561 | 23.83584455 |
| 10 | 50 | 40 | 24.49557521 | 48.77685996 | 26.73563265 |
| 45 | 55 | 0 | 22.4905808 | 53.48652661 | 24.02944855 |
| 75 | 0 | 25 | 73.91311353 | 0.030814477 | 25.89483813 |
| 75 | 15 | 10 | 41.11756881 | 10.38309322 | 48.48435688 |
| 70 | 10 | 20 | 40.90424064 | 10.33828069 | 48.74163516 |
| 45 | 40 | 15 | 27.83078409 | 40.06035673 | 32.1183135 |
| 60 | 0 | 40 | 60.43484954 | -0.162744519 | 39.63114913 |
| 5 | 20 | 75 | 8.676806572 | 19.05772438 | 72.16627528 |
| 30 | 35 | 35 | 28.91706623 | 36.91427273 | 34.17817407 |
| 10 | 35 | 55 | 28.83877681 | 37.13026324 | 34.04045499 |
| 85 | 5 | 10 | 40.85761909 | 10.32893331 | 48.79741523 |
| 30 | 15 | 55 | 41.20470755 | 10.40322135 | 48.37743945 |
| 15 | 5 | 80 | 40.86123723 | 10.32965863 | 48.79308643 |
| 65 | 0 | 35 | 65.81077754 | -0.100856041 | 34.02657307 |
| 0 | 30 | 70 | 31.15420024 | 31.63399001 | 37.22297721 |
| 40 | 35 | 25 | 28.9572664 | 36.80461916 | 34.2476383 |
| 30 | 10 | 60 | 40.91671842 | 10.34078478 | 48.72670383 |
| 55 | 45 | 0 | 26.5440115 | 43.62574465 | 29.83942217 |
| 25 | 75 | 0 | 11.87762645 | 76.52205196 | 11.59645371 |
| 0 | 90 | 10 | 5.74870533 | 89.32850816 | 4.912267476 |
| 60 | 20 | 20 | 62.71388634 | 20.12095409 | 17.16941198 |
| 45 | 35 | 20 | 28.97903022 | 36.7456144 | 34.28488557 |
| 10 | 40 | 50 | 27.70812521 | 40.41511023 | 31.88621161 |
| 35 | 40 | 25 | 27.79785558 | 40.1558813 | 32.05571585 |
| 20 | 40 | 40 | 27.7421117 | 40.31711404 | 31.95022372 |
| 5 | 85 | 10 | 7.481735776 | 85.71889482 | 6.790745215 |
| 80 | 5 | 15 | 40.85782175 | 10.32897395 | 48.79717276 |
| 10 | 65 | 25 | 16.95083078 | 65.74161138 | 17.30896784 |
| 0 | 50 | 50 | 24.43528241 | 48.92219287 | 26.65055181 |
| 50 | 25 | 25 | 31.77832968 | 24.62960595 | 43.59662125 |
| 60 | 10 | 30 | 40.90716844 | 10.33886806 | 48.73813185 |
| 10 | 0 | 90 | 6.45063831 | 0.688974286 | 93.43528231 |
| 75 | 5 | 20 | 40.85803297 | 10.32901629 | 48.79692005 |
| 25 | 10 | 65 | 40.91845965 | 10.34113441 | 48.72462002 |
| 30 | 40 | 30 | 27.77847024 | 40.21202053 | 32.01896091 |
| 5 | 5 | 90 | 40.86191163 | 10.32979383 | 48.79227957 |
| 60 | 15 | 25 | 41.14239311 | 10.38873323 | 48.4539924 |
| 90 | 0 | 10 | 92.67244831 | 0.335814851 | 8.120659032 |
| 20 | 70 | 10 | 14.35438198 | 71.29068897 | 14.35367823 |
| 30 | 5 | 65 | 40.86030252 | 10.32947126 | 48.79420474 |
| 35 | 35 | 30 | 28.93573117 | 36.86325352 | 34.21053322 |
| 10 | 70 | 20 | 14.27435893 | 71.46052334 | 14.26378339 |
| 5 | 0 | 95 | 1.808586968 | 1.21630535 | 97.44115414 |
| 25 | 70 | 5 | 14.39436076 | 71.20581817 | 14.39861196 |
| 5 | 60 | 35 | 19.62602697 | 59.918108 | 20.45988098 |
| 80 | 0 | 20 | 78.85410303 | 0.134398015 | 21.27610358 |
| 40 | 5 | 55 | 40.85973264 | 10.32935702 | 48.79488654 |
| 5 | 25 | 70 | 32.71749146 | 24.81820863 | 42.47262704 |
| 5 | 65 | 30 | 16.91319594 | 65.82265972 | 17.26551656 |
| 50 | 40 | 10 | 27.84982 | 40.00504193 | 32.15459318 |
| 10 | 55 | 35 | 22.23386331 | 54.07457028 | 23.69790963 |
| 95 | 5 | 0 | 40.85722824 | 10.32885493 | 48.79788288 |
| 30 | 60 | 10 | 19.8238243 | 59.48173246 | 20.69864446 |
| 50 | 45 | 5 | 26.52280236 | 43.6818366 | 29.8045314 |
| 85 | 15 | 0 | 41.102783 | 10.37977546 | 48.50240091 |
| 55 | 15 | 30 | 41.15129223 | 10.39077489 | 48.44308736 |
| 0 | 70 | 30 | 14.19425149 | 71.63047563 | 14.17385486 |
| 10 | 60 | 30 | 19.65916299 | 59.84506946 | 20.49981463 |
| 50 | 30 | 20 | 31.87127836 | 30.06350621 | 38.07706933 |
| 20 | 20 | 60 | 16.68894121 | 19.04962992 | 64.17563803 |
| 40 | 20 | 40 | 39.29382258 | 19.54296023 | 41.12124769 |
| 65 | 10 | 25 | 40.90568389 | 10.33857022 | 48.73990823 |
| 65 | 25 | 10 | 31.35049437 | 24.54395587 | 44.1083894 |
| 15 | 50 | 35 | 24.52881037 | 48.69662871 | 26.78265103 |
| 75 | 25 | 0 | 31.03284244 | 24.48036608 | 44.48835612 |
| 35 | 45 | 20 | 26.4497008 | 43.87460508 | 29.68483628 |
| 70 | 5 | 25 | 40.85825353 | 10.32906051 | 48.79665617 |
| 35 | 5 | 60 | 40.86001448 | 10.32941352 | 48.79454934 |
| 20 | 60 | 20 | 19.74719428 | 59.65090347 | 20.60603183 |
| 50 | 10 | 40 | 40.91021334 | 10.33947904 | 48.7344883 |
| 55 | 5 | 40 | 40.85895211 | 10.32920055 | 48.79582038 |
| 50 | 15 | 35 | 41.16117019 | 10.39305254 | 48.43097139 |
| 5 | 80 | 15 | 9.462030199 | 81.58576874 | 8.945731947 |
| 40 | 40 | 20 | 27.81436713 | 40.10800743 | 32.087079 |
| 65 | 5 | 30 | 40.8584794 | 10.32910579 | 48.79638594 |
| 15 | 10 | 75 | 40.92202558 | 10.34185065 | 48.7203523 |
| 35 | 50 | 15 | 24.65341654 | 48.3950493 | 26.95970687 |
| 55 | 35 | 10 | 29.02049495 | 36.63390051 | 34.35514767 |
| 35 | 65 | 0 | 17.16317354 | 65.28392788 | 17.55452125 |
| 60 | 25 | 15 | 31.50437544 | 24.57476121 | 43.92432063 |
| 30 | 65 | 5 | 17.12581657 | 65.36449614 | 17.51127261 |
| 35 | 15 | 50 | 41.19284583 | 10.4004312 | 48.39204392 |
| 45 | 10 | 45 | 40.91177329 | 10.3397921 | 48.73262162 |
| 65 | 35 | 0 | 29.06288563 | 36.52064349 | 34.42602848 |
| 25 | 20 | 55 | 21.36649805 | 19.12759914 | 59.42889898 |
| 95 | 0 | 5 | 97.56705089 | 0.213606132 | 2.853860923 |
| 35 | 30 | 35 | 31.64324443 | 30.56324143 | 37.80515014 |
| 50 | 50 | 0 | 24.7435361 | 48.17615377 | 27.08854157 |
